# Exosome Derived from Human Umbilical Cord Mesenchymal Cell Exerts Immunomodulatory Effects on B Cells from SLE Patients

**DOI:** 10.1155/2023/3177584

**Published:** 2023-05-12

**Authors:** Ying Zhao, Wenbin Song, Zilin Yuan, Min Li, Gang Wang, Liping Wang, Yueping Liu, Bo Diao

**Affiliations:** ^1^School of Medicine, Wuhan University of Science and Technology, Wuhan 430065, Hubei Province, China; ^2^Basic Medical Laboratory, General Hospital of the Central Theater Command, Wuhan 430070, Hubei Province, China; ^3^Hubei Key Laboratory of Central Nervous System Tumor and Intervention, Wuhan 430070, Hubei Province, China; ^4^People's Hospital of Xinyang, Xinyang 464000, Hennan Province, China

## Abstract

**Background:**

Excessive proliferation and activation of B cells, resulting in the production of various autoantibodies, is a crucial link and significant feature of the pathogenesis of systemic lupus erythematosus (SLE), as well as the pathological basis of systemic multiorgan damage. However, whether exosomes derived from human umbilical cord mesenchymal stem cells (hucMSCs-Exo) are involved in the immune regulation of SLE has not been clarified.

**Objectives:**

Therefore, our study aimed to investigate the efficacy of hucMSCs-Exo for treating SLE.

**Methods:**

hucMSCs-Exo and peripheral blood mononuclear cells (PBMCs) of SLE patients were cocultured *in vitro*, and B cell apoptosis, activation, proliferation, and inflammation levels were detected by flow cytometry. Subsequently, the expression level of miR-155 in B lymphocytes of SLE patients was detected by qRT-PCR, and the target gene relationship between miR-155 and SHIP-1 was found through bioinformatics and dual luciferase activity experiments, which verified the inhibition of miR-155 in B lymphocytes of SLE patients to regulate immunity.

**Results:**

We found that hucMSCs-Exo promoted B cell apoptosis, prevented B cell overactivation, and reduced inflammation. MicroRNA-155 (miR-155) has a powerful regulatory function in B cells. It was demonstrated that hucMSCs-Exo acts synergistically with miR-155 inhibitors to target SHIP-1 to B cells more effectively than exosomes alone.

**Conclusion:**

Our results provide insight into how hucMSCs-Exo regulates autoimmunity in patients with lupus and suggest targeting miR-155 for autoimmunity while protecting immunity.

## 1. Introduction

Systemic lupus erythematosus (SLE) is a chronic systemic autoimmune disease of unknown etiology, affecting predominantly women of reproductive age [1]. Conventional SLE therapies, such as immunomodulatory agents and immunosuppressive drugs, remain limited, and the inflammatory milieu is only temporarily effectively controlled when the lupus disease is under flaring condition. Long-term immunosuppression is often required; however, these immunosuppressive drugs can potentially induce infection, secondary malignancy, and organ failure [2]. Therefore, finding effective therapeutic options with mild side effects and enduring immunosuppressive effects is urgent.

Due to the immunomodulatory potential, human umbilical cord mesenchymal stem cells (hucMSCs), extracted from donor sources through noninvasive procedures, exerting immunosuppressive and hematopoiesis-supportive effects [3], have been increasingly investigated in the context of SLE and other immunologic diseases [4]. They can block specific effector cells in SLE [5], the generation of regulatory T cell subset, and release various anti-inflammatory cytokines, including interleukin-10 (IL-10) and transforming growth factor (TGF) *β*1 [6], indicating that hucMSCs are a promising approach to controlling SLE disease. However, this creates a concern about a potential tumorigenic effect after MSC implantation [7]. Compared with hucMSCs-based therapy, human umbilical cord mesenchymal stem cell-derived exosomes (hucMSCs-Exo) based cell-free treatment has the advantages of nontumorigenicity, high stability, and no vascular obstruction [8]. Up to now, whether and how hucMSCs-Exo regulates SLE immune response is still unknown.

Recent studies have shown that exosomes exert critical regulatory effects on the physiological functions of recipient cells via regulating miRNAs [9]. To date, miR-155, a highly conserved miRNA among mammals serving as a critical regulator of cell proliferation [10], is one of the most highly implicated miRNAs in autoimmunity. Available data demonstrated that miR-155 has a robust regulatory function in B cells [11]. SLE's immune response hallmark is the autoantibodies' production by autoreactive B cells reacting to self-antigens and triggering an overwhelming inflammatory response, resulting in multifaceted immune modulation, including deficiency and hyperactivity of the immune system [12]. Although the therapeutic efficacy of hucMSCs-Exo has not been discussed in the context of SLE, the anti-inflammatory, immunomodulatory, and other potentials of hucMSCs-Exo have been demonstrated in many other diseases. Therefore, it is reasonable to hypothesize that hucMSCs-Exo may attenuate inflammation and regulate the immune response of B cells in SLE patients by regulating miR-155 and its target gene.

To confirm our hypothesis, we first demonstrated the immunomodulatory effects of hucMSCs-Exo on SLE patients' B cell proliferation, apoptosis, activation, and inflammation in an in vitro cell experiment. Then, we explore and characterize the underlying biological mechanism of the immunomodulatory effect of hucMSCs-Exo through coculture inhibition of miR-155 and hucMSCs-Exo.

## 2. Materials and Methods

### 2.1. Patients and Controls

Twenty-eight SLE patients with no history of corticosteroids or immunosuppressive drug use were recruited from the General Hospital of the Central Theater Command from January 2022 to May 2022. All the enrolled SLE patients had not taken glucocorticoids or immunosuppressive agents when they came to our hospital for diagnosis and treatment. The SLE patients fulfilled the 1997 SLE classification criteria revised by the American College of Rheumatology [13]. Twenty-two ethnically, age- and sex-matched healthy controls (HC), who had no history of autoimmune or inflammatory diseases themselves and in their families, were also recruited into the present study. All participants signed written informed consent, and the Ethics Committee approved the study of the General Hospital of Central Theater Command (approval no. [2021]065-02).

### 2.2. Sample Collection and Preparation

Peripheral blood of SLE patients and healthy controls were collected. The blood of SLE patients was collected prior to therapy with glucocorticoids and immunosuppressive agents. Diluted with equal proportion phosphoric acid buffered brine (PBS). Peripheral blood mononuclear cells (PBMCs) were prepared by Ficoll-Plaque (MultiSciences Biotech, China) density gradient centrifugation (2,000 rpm, 23°C, 30 min), washed in PBS (Biosharp, China) twice, and then resuspended in RPMI 1640 culture medium (Gibco, USA) at a concentration of 2 × 10^6^ cells/mL. Further, 10% fetal bovine serum Premium (PAN FBS, Germany) and 1% penicillin/streptomycin were supplemented with cells at 37°C with 5% CO_2_.

A typical panel of markers used to identify the significant subset of lymphocytes included CD3 (clone: UCHT1), CD4 (clone: RPA-T4), CD8 (clone: RPA-T8), CD19 (clone: HIB19), and CD16 + 56 (clone: 3G8). The isolated PBMCs were resuspended with appropriate amount of PBS. For the staining of surface antigens, cells were incubated with FITC-conjugated anti-CD3, APC-conjugated anti-CD4, PE-conjugated anti-CD8, APC-conjugated anti-CD19, and PE-conjugated anti-CD16 + 56 (all from BioLegend Bioscience, USA). Mouse anti-human FITC-, PE-, and APC-conjugated IgG1 were used as isotype controls. The samples were detected by flow cytometry (Agilent NovoCyte, Model number: D2040, China) and analyzed by NovoExpress software. The supernatant of PBMC was taken, and IL-6, IL-10, IFN-*γ*, IL-17, IL-4, and TNF-*α* were detected by flow cytometry with multiple microspheres according to the manufacturer's instructions and analyzed by LEGENDplex software.

### 2.3. Preparation and Identification of hucMSCs

hucMSCs were provided by the Basic Medical Laboratory of the General Hospital of the Central Theater Command. hucMSCs at passage 3 were resuscitated, then cultured in *α*-MEM complete medium (10% fetal bovine serum + 1% double antibody + *α*-MEM basal medium), incubated in 5% CO_2_ and 37°C. The protocol for preparation and identification of huMSCs was published previously [14]. Briefly, cells were observed under an inverted microscope and were in good condition. The cells were collected and centrifuged, and the concentration of cells was adjusted to 1 × 10^6^ cells/mL in PBS. According to Biolegend instructions, hucMSCs labeled with flow cytometry were stained for immunophenotype characterization. The antibody contains three positive markers (FITC-CD73, PE-CD90, and FITC-CD105) and five negative cocktails (APC-CD34, PerCP-CD45, PE-CD11b, APC-CD19, and PerCP-HLA-DR) [14], as well as their homotype controls. Analysis was performed in a flow cytometer (Agilent NovoCyte, China). hucMSCs at passage 3 were cultured in stem cell osteogenic and adipogenic differentiation media (Stemcell, Canada) to identify the differentiation properties. To identify differentiation characteristics, hucMSCs of the third generation were cultured in stem cell osteogenic and adipogenic differentiation medium (Stemcell, Canada) for 23 days according to the manufacturer's protocol. hucMSCs were fixed and dyed with Alizarin red for osteogenic cells and Oil red for adipose cells. Cells cultured in normal medium served as controls.

### 2.4. Extraction and Identification of hucMSCs-Exo

The hucMSCs were cultured with a fusion rate of 60%–70%. After being replaced with serum-free MEM medium (*α*-MEM, TBD, China) for 48 hr, the cell supernatant was collected and centrifuged at 300 g successively for 10 min, another 10 min at 2,000 g, followed by 30 min at 20,000 g, all at 4°C. Finally, the supernatant was centrifuged at high speed at 120,000 g for 70 min at 4°C. The precipitate was resuspended with aseptic PBS buffer solution at 4°C, then centrifuged at 120,000 g for 70 min, and an appropriate amount of aseptic PBS was resuspended, filtered by a 0.22 *μ*M filter, and frozen at −80°C for later use.

Transmission electron microscope was used to observe the characteristical morphologies of the exosomes. (HITACHI Transmission Electron Microscope HT7700). The distribution of particle size of hucMSCs-Exo was analyzed using a nanoparticle tracking analysis system (Particle Metrix, Meerbusch, Germany). Surface markers of exosomes, including CD63 (PE, Biolegend, USA) and CD81 (APC, Biolegend, USA), were detected by flow cytometry.

### 2.5. Coculture of PBMCs with hucMSCs or hucMSCs-Exo

hucMSCs were plated in six-multiwell flat bottom culture plates (NEST, China) at 5 × 10^4^ cells/well density and cultured in basal medium supplemented with FBS (10%). After 6 hr for cell adhesion, the medium was aspirated and replaced with fresh PBMCs at 5 × 10^5^ cells/well, corresponding to a ratio of hucMSCs/PBMC 1 : 10. To assess apoptosis, PBMCs were collected after coculture using Annexin V-FITC/PI apoptosis kit (MULTI Sciences, China) according to the manufacturer's instructions, centrifuged at 1,500 rpm for 5 min, washed with PBS, and resuspended in 500 *μ*L 1 × binding buffer. B cells were sorted by sequentially adding 5 *μ*L CD19 (APC, Biolegend, USA), then incubated with FITC and PI for 15 min at room temperature and then analyzed by flow cytometry (Agilent NovoCyte, Model number: D2040, China).

The concentration of purified hucMSCs-Exo was measured via BCA protein concentration Assay Kit (Solarbio, China). To select the optimal concentration hucMSCs-Exo for the subsequent experiments, a series of concentrations of hucMSCs-Exo, including 20, 50, 100, and 200 *μ*g/mL, was prepared. The optimal concentration was determined according to the minimum concentration that could promote cell apoptosis.

### 2.6. Cell Apoptosis, Cell Proliferation Assays, and B Cell Activation Detection Assays

Cell apoptosis was detected by Annexin V-FITC/PI apoptosis kit, flow cytometry, and NovoExpress analysis. The apoptosis rate of B cells was detected by flow cytometry. PBMCs were isolated from SLE patients, and after coculture with hucMSCs and hucMSCs-Exo for 48 hr, PBMCs were collected. Experimental cells were prepared into cell suspension, and B cells were labeled with CD19 (APC, Biolegend, USA). Cells were stained and labeled with Annexin V-FITC/PI according to the kit instructions, detected by flow cytometry, and analyzed by Agilent NovoCyte flow cytometer and NovoExpress software.

To assess the proliferative stimulatory activity of hucMSCs and their exosomes, we labeled 1 × 10^6^ PBMCs with cell tracking CFSE (Beyotime Biotechnology, China), which were cocultured with hucMSCs or hucMSCs-Exo isolated from passages 3, 6, and 9, as described in the manufacturer's instructions. These PBMCs were isolated from SLE participants and had no further stimulation with anti-CD3 and anti-CD28. In addition to healthy controls, all PBMCs were collected after 72 hr and analyzed by Agilent NovoCyte flow cytometer and NovoExpress software.

PBMC was cocultured with huc-MSCs and hucMSCs-Exo for 0, 4, 12, 24, and 36 hr, and PBMC was collected to evaluate B cell activation. CD19- (APC, Biolegend, USA) labeled B cells and CD69 (PE, Biolegend, USA) were used to detect B cell activation, which was protected from light for 15 min, and analyzed by flow cytometry and NovoExpress software.

### 2.7. RNA Extraction and qPCR Analysis

Total RNA was extracted from PBMCs using Trizol Reagent (TaKaRa, Japan). First, proper amount of chloroform was added and centrifuged. A new enzyme-free EP tube was taken out and 400 *μ*L isopropyl alcohol was added. After centrifugation, proper amount of RNA from the upper layer was taken and added to it. RNA was reverse-transcribed into cDNA using Thermo Scientific RevertAid RT kit (Thermo Scientific, USA). qRT-PCR was performed using TB Green Premix Ex Taq II (Tli RNaseH Plus, TaKaRa, Japan), followed by testing using the LightCycler 96 PCR instrument (Roche, Tusem, China). U6 or GAPDH was used as internal parameters, and the relative expression levels of miRNAs or mRNAs were calculated by 2^−*ΔΔ*Ct^. Primer sequences were acquired from BIG Tech Solutions (Beijing Liuhe, China) (Table 1).

### 2.8. Cell Transfection

PBMCs were transfected with the indicated miRNA antagomirs or NCs using Lipofectamine 2000 (Invitrogen, Carlsbad, CA, USA) following the manufacturer's instructions. Twenty-four hours after transfection, cells were collected for subsequent coculture with hucMSCs-Exo to detect B cell apoptosis, proliferation, activation, and cytokines. Antagomir-155 and antagomir-NC were designed and synthesized by RiboBio Biotechnology (Guangzhou, China).

### 2.9. Plasmid Construction and Luciferase Reporter Assay

The possible miRNA-binding sites on the SHIP-1 were predicted using the TargetScan, miRDB, and miRWalk databases. Briefly, the miR-155-binding sites in the SHIP-1 3′-UTR sequence were cloned into a psiCHECK-2 vector (YouBiao, Hunan, China). For the luciferase reporter assay, 293T cells were cotransfected with the luciferase reporter plasmids combined with miR-155 mimics or controls, respectively. After 24 hr of transfection, the cells were lysed and measured for luciferase activities using the chemiluminescence immune analyzer (Compson, Shijiazhuang, China) following the manufacturer's protocols.

### 2.10. Western Blotting Analysis

Cultured cells were collected and centrifuged for 5 min at 1,500 rpm, cell lysate containing protease and phosphorylase (Beyotime, China) was added, and total protein was extracted by lysis on ice. Sample handling and concentration determination were carried out according to the instructions in the BCA protein Quantification kit (Beyotime, China). After protein denaturation, glue making, load sample, separated by electrophoresis, transferred to PVDF membrane (Millipore, MA, USA), and blocked with 5% skim milk powder. For primary antibodies, antibody *β*-tubulin was diluted 1 : 50,000, and SHIP-1, p-ERK, and ERK were diluted 1 : 1,000 according to the manufacturer's instructions and incubated with membranes overnight at 4°C. Next, the cells were incubated with the corresponding secondary antibody (HRP-conjugated goat anti-rabbit antibodies, 1 : 600, Beyotime, A0208).

### 2.11. Statistical Analysis

Data were analyzed using GraphPad Prism Version 8.0. All data were presented as mean ± standard deviation (SD). Differences between the two data sets were statistically analyzed using the Student *t*-test while comparisons between multiple groups were done by one-way analysis of variance. Statistically significant level was set at *P* value <0.05.

## 3. Results

### 3.1. Absolute Numbers of CD19+ B Cells Increased in SLE Patients

**Figure 1:**
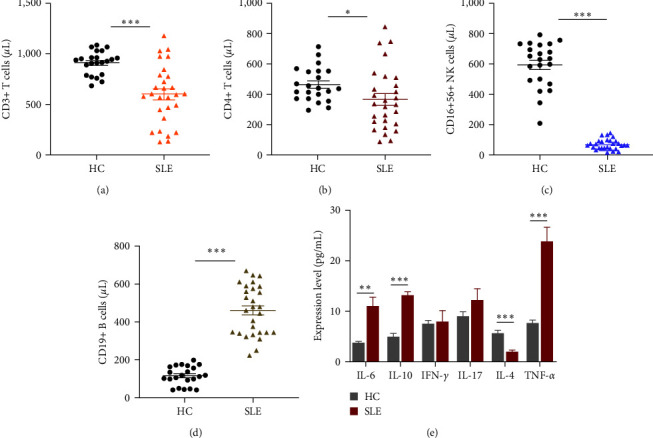
Clinical characteristics. Distribution of (a) CD3+ T, (b) CD4+ T, (c) CD16 + 56 NK, and (d) CD19+ B cells in SLE patients compared with healthy controls. (e) Compared with healthy controls, the expression of cytokines in the supernatant of SLE peripheral blood mononuclear cells (PBMCs) was detected. (*n* = 28);  ^*∗*^*P* < 0.05,  ^*∗∗*^*P* < 0.01,  ^*∗∗∗*^*P* < 0.001.

A total of 28 patients diagnosed with SLE and 22 healthy controls were included. Compared with healthy controls, the absolute numbers of CD3+ T cells (Figure 1(a)), CD4+ T cells (Figure 1(b)), and NK cells (Figure 1(c)) were significantly downregulated. In contrast, the absolute numbers of CD19+ B cells (Figure 1(d)) increased in SLE patients, and the levels of IL-6, 1L-10, and TNF-*α* in SLE patients significantly increased. The levels of IL-4 decreased (all *P* < 0.05) (Figure 1(e)). The demographic information and some other clinical manifestations of enrolled participants are also detailed in Table 2.

### 3.2. Isolation and Identification of hucMSC and hucMSCs-Exo

**Figure 2:**
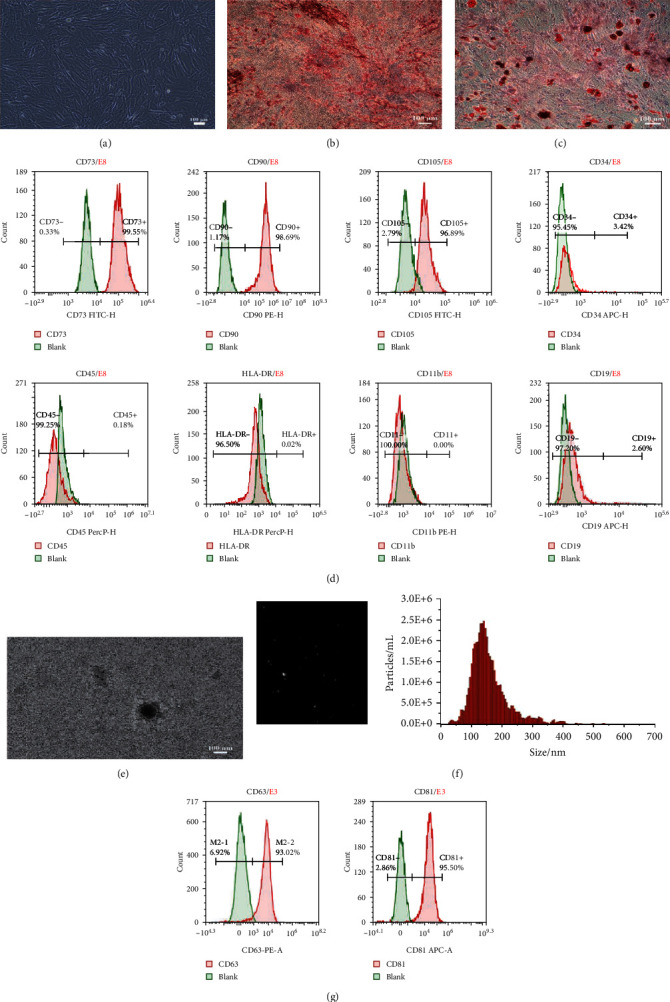
Isolation and identification of hucMSCs and hucMSC-Exo. (a, b) Osteogenic differentiation of hucMSCs was performed in the differentiation medium, while the control cells were grown in a regular medium. Both were dyed with Alizarin Red S staining. Most hucMSCs were Alizarin red-positive. (c) Oil red O staining was used to stain hucMSCs after adipogenic differentiation, showing the Oil red O-positive lipid droplets (bar = 100 *μ*m). (d) Flow cytometry analysis of the phenotypic markers of hucMSCs showed that hucMSCs were positive for CD73, CD90, and CD105 and negative for CD34, CD45, and human leukocyte antigen (HLA)-DR. (e) Transmission electron microscopic images of typical hucMSC-Exo. Scale bar = 100 nm. (f) The size distribution of the hucMSC-Exo was determined using a nanoparticle tracking analysis. hucMSC-Exo had an original concentration of 2.0e + 10 particles/mL, a mean size of 145.1 nm with a peak of 137.7 nm. (g) Expression of positive markers, CD63 and CD81, was mainly expressed in hucMSC-Exo using flow cytometry analysis.

hucMSCs were cultured for 2 weeks after being successfully isolated. hucMSCs around the tissue block showed fibroblast-like morphology and were attached to the surface of the culture flask. After passage, hucMSCs proliferated and arranged in a spiral pattern (Figure 2(a)). Osteogenic and adipogenic differentiation of hucMSCs were confirmed using alizarin red staining (Figure 2(b)) and Oil red O staining (Figure 2(c)). Flow cytometry results demonstrated that the positive markers, including CD73, CD90, and CD105, were highly expressed. Furthermore, the negative markers (CD11b, CD19, CD34, CD45, and HLA-DR) were not expressed (Figure 2(d)).

The supernatant of the third to seventh passages of hucMSC-Exo was selected and purified by continuous ultrahigh-speed centrifugation. hucMSCs-Exo is purified from the serum-free supernatant of hucMSCs by continuous ultrahigh-speed centrifugation. Transmission electron microscope results showed spherically shaped vesicles within a size range of exosomes (Figure 2(e)). The sizes of exosomes were characterized by a nanoparticle tracking analysis system which tracks the dynamics of particle movement and approximates it with Brownian motion to obtain size information. The average size of exosomes was approximately 130 nm in diameter (Figure 2(f)). CD63 and CD81, conventional exosome markers, were significantly expressed in isolated hucMSCs-Exo (Figure 2(g)).

### 3.3. hucMSCs-Exo Promoted B Cell Apoptosis, Inhibited Proliferation, and Prevented Overactivation and Inflammation

To assess the effects of hucMSCs-Exo on B cells *in vitro*, we cocultured PBMC of SLE patients with hucMSCs-Exo, then sorted B cells by CD19 after coculture. Although this study focused on exosomes, hucMSCs were also included as a control group to compare the effects of hucMSCs-Exo and hucMSCs.

**Figure 3:**
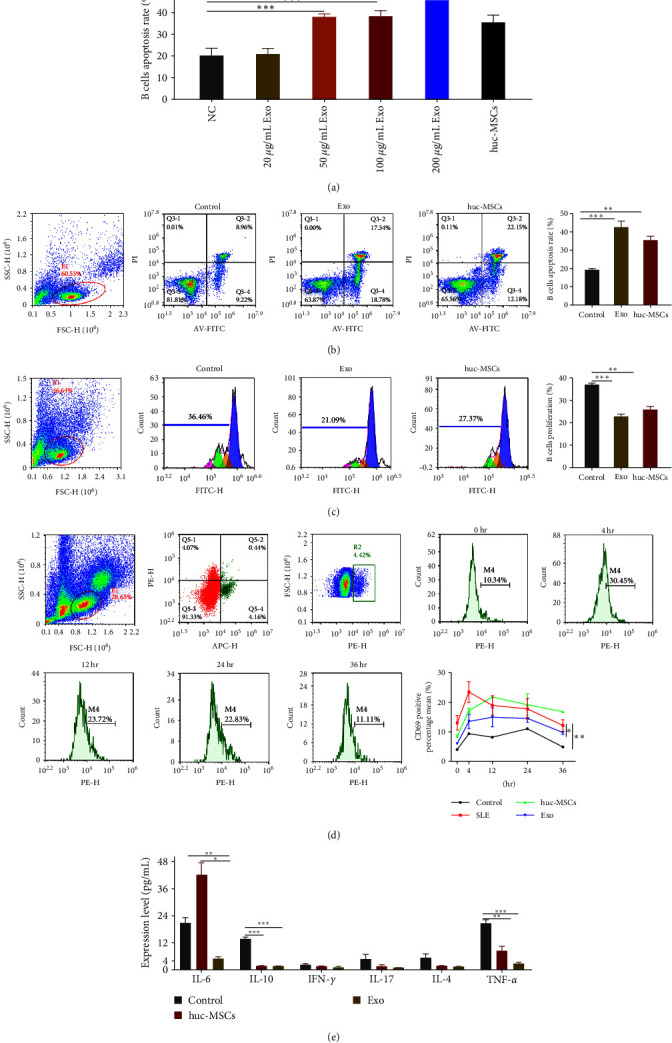
hucMSCs-Exo promoted B cell apoptosis, inhibited proliferation, and prevented overactivation and inflammation. (a) Different concentrations of Exo were cocultured with B cells from SLE. (b) B cell apoptosis was determined using Annexin V-FITC kit (c) B cell proliferation was determined using the CFSE kit. (d) The expression of B cell activation marker CD69. (e) Cytokine expression levels after coculture with hucMSCs and exosomes compared with control. Exosomes were isolated from hucMSC. Data are expressed as the mean ± SEM.  ^*∗*^*P* < 0.05,  ^*∗∗*^*P* < 0.01,  ^*∗∗∗*^*P* < 0.001.

The used dose of exosomes in most studies is approximately 10–100 *μ*g/mL. However, the concentration used in our subsequent experiments is 50 *μ*g/mL based on the following results (Figure 3(a)). There was no significant difference between the lower dose (20 *μ*g/mL) and untreated PBMC (*P* > 0.05). No significant difference was observed in the inhibitory effect of 50 and 100 *μ*g/mL doses on B cell apoptosis. Additionally, doses higher than 100 *μ*g/mL were impractical because of inadequate exosome preparation.

The proapoptotic rate of B cells in the hucMSCs-Exo group was the highest, followed by the hucMSC and untreated B cell groups (Figure 3(b)). Flow cytometry showed that hucMSCs and hucMSCs-Exo could promote B cell proliferation compared with the control group. Still, hucMSCs-Exo had a better effect in promoting B cell proliferation (Figure 3(c)).

To analyze the hyperactivation of B cells, we cocultured PBMC with hucMSC and hucMSCs-Exo. B cells were sorted by flow cytometry with CD19, followed by evaluation of B cell activation with CD69. Compared with healthy controls, B cells in SLE patients peaked at hour 4 and gradually decreased until 36 hr, whereas the exosome group reduced B cell hyperactivation (Figure 3(d)). The cytokines IL-6, IL-10, and TNF-*α* are known to be elevated in SLE patients, which is also confirmed in Figure 1. After coculture, the levels of cytokines IL-16, IL-10, INF-*γ*, IL-17, 1L-4, and TNF-*α* were detected by flow cytometry. Compared with the control group, IL-16, IL-10, and TNF-*α* levels significantly decreased in the exosome group (Figure 3(e)). In contrast, the level of IL-6 in the huc-MSCs group was significantly higher than in the control group. It may be that huc-MSCs themselves secrete IL-6 when paracrine, leading to a significant increase in IL-6 levels after coculture.

### 3.4. MiR-155 Targets SHIP-1

**Figure 4:**
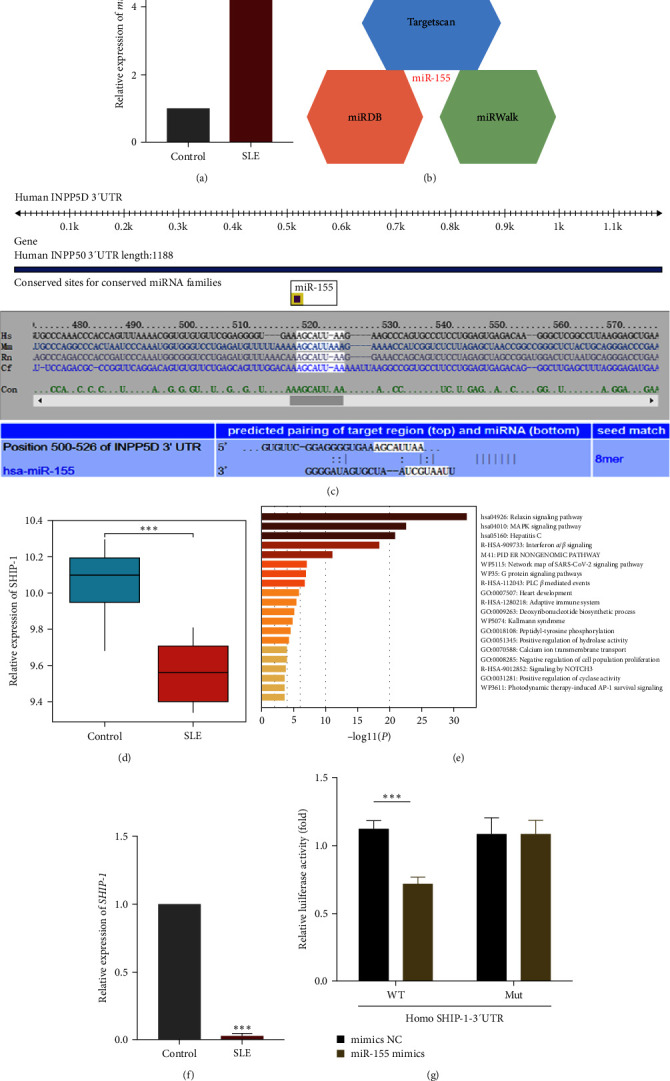
miR-155 targets SHIP-1. (a) qPCR was used to detect the expression of miRNA-155 in B cells of the control group and SLE patients (*n* = 3 per group). U6 snRNA was used as an endogenous control. (b) The potential targets of miR-155 were predicted by integrating the results of three databases (TargetScan, miRDB, and miRWalk). (c) Conservation of the miR-155 target sequence in SHIP1 3′-UTR among different species and conservation of the miR-155 sequence among different species. (d) GEO chip GSE4588 analyzed the expression of SHIP-1. (e) The SHIP-1-related genes were screened for GO analysis and showed to be related to MAPK/ERK pathway. (f) The SHIP-1 expression was determined by qPCR (*n* = 3 per group). GAPDH snRNA was used as an endogenous control. (g) The dual-luciferase reporter assay was performed in 293 T cells. Cells were cotransfected with the wild- or mutant-type SHIP-1, 3′-UTR luciferase reporter plasmids, and miR-155 mimics or mimics-NC. The ratio of Renilla activity: Firefly activity represents luciferase activity. Data are expressed as the mean ± SEM.  ^*∗*^*P* < 0.05,  ^*∗∗∗*^*P* < 0.001.

To investigate whether miR-155 is involved in SLE immune response, we used qPCR to detect its expression. We found that miR-155 expression significantly increased in SLE patients' B cells compared with healthy controls (Figure 4(a)).

To illustrate the role of miR-155 in the pathogenesis of SLE, the TargetScan, miRDB, and miRWalk databases were used to predict the target genes of miR-155 (Figure 4(b)). Subsequently, we analyzed the possible targets of miR-155 in cells to reveal the molecular mechanism of miR-155 affecting the apoptosis, proliferation, and activation of B cells. The bioinformatics analysis revealed that the 3′-UTR of SHIP-1 (the SH2 domain-containing inositol 5′-phosphatase 1) mRNA contains a complementary binding site in the seed region of miR-155, which is conserved across species (Figure 4(c)). Second, the expression of SHIP-1 was analyzed by GEO chip GSE4588 (Figure 4(d)). The genes related to SHIP-1 were screened for GO analysis, which showed they were related to the MAPK/ERK pathway (Figure 4(e)). We examined its expression using qPCR and found that mRNA SHIP-1 expression significantly increased in SLE patients' B cells compared with healthy controls (Figure 4(f)).

We carried out luciferase reporter assays to provide direct evidence that SHIP-1 is a target of miR-155. The results showed that miR-155 mimics significantly decreased the luciferase activity for wild-type 3′-UTR of SHIP-1 but did not inhibit the mutated 3′-UTR of SHIP-1 (Figure 4(g)). In conclusion, miR-155 inhibits SHIP-1 expression in B cells by directly targeting the 3′-UTR of SHIP-1 mRNA.

### 3.5. MiR-155 Modulates SHIP-1 Expression and the Downstream ERK Kinase Pathway after BCR and Fc*γ*RIIB Coligation

**Figure 5:**
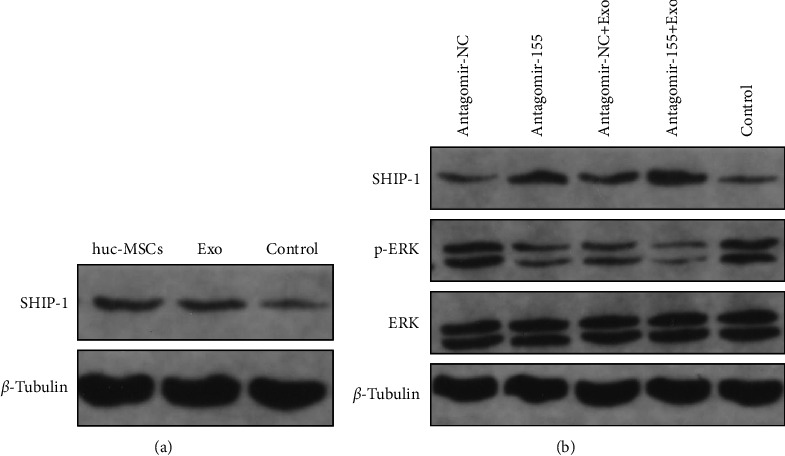
MiR-155 modulates SHIP-1 expression and the downstream ERK kinase pathway after BCR and Fc*γ*RIIB coligation. (a) Western blot analysis was used to detect the protein level of SHIP-1 after coculture with hucMscs and exosomes. *β*-tubulin was used as an internal control. (b) Western blot analysis was used to detect the protein level of SHIP1 after transfection with agomir-155 or agomir-NC after intervention with hucMSCs-Exo. *β*-Tubulin was used as an internal control. After incubation with hucMSCs-Exo and transfection with agomir-155 or agomir-NC, whole-cell lysates were probed with antiphosphorylated ERK, stripped, and reprobed with anti-total ERK. *β*-Tubulin was used as an internal control. Ratio: p-ERK/total ERK determined by Bio-Rad Quantity One software.

The SH2 domain-containing inositol 5′-phosphatase 1 (SHIP-1) phosphatase acts downstream of inhibitory cell-surface receptors, including the IgG inhibitory receptor Fc*γ*RIIB (Fc*γ*RIIB), which is essential in opposing B-cell activation signals in mice and humans [15]. After collating the Fc*γ*RIIB with the B-cell receptor (BCR), Fc*γ*RIIB recruits SHIP-1 to the plasma membrane, which negatively regulates cell survival, Ca^2+^-dependent effector functions, and ERK activation, thus controlling cell proliferation, anergy, and apoptosis [16, 17]. MiR-155 has been reported to regulate SHIP-1 expression in mammalian myeloid and malignant B cells [18]. At present, it is unclear whether the regulation of SHIP-1 by miR-155 affects the autoimmune response of SLE. We found that the level of miR-155 was significantly increased in the B cells of SLE patients. As detected by Western blotting, the SHIP-1 level in B cells treated with hucMSCs and Exo was significantly higher than that in the untreated control group (Figure 5(a)). We also found that miR-155-inhibited B cells had a 1.7-fold increase in SHIP-1 levels after coordinated hucMSCs-Exo intervention (Figure 5(b)). The above results indicated that SHIP-1 protein expression was low in unstimulated B cells, possibly because miR-155 was highly expressed in B cells.

In contrast, inhibition of miR-155 in B cells combined with hucMSCs-Exo intervention significantly increased SHIP-1 levels in B cells. Therefore, inhibition of miR-155 can restore the expression level of SHIP-1 in B cells of SLE patients. In this context, miR-155 is at least partly responsible for the low expression of SHIP-1 in B cells. The results also showed that inhibition of miR-155 in B cells maintained SHIP-1 at a high level, thus making B cells less susceptible to activation.

Next, we investigated how ERK activation was affected by dysregulation of SHIP-1 expression in miR-155-inhibited B cells. P-ERK activation was inhibited in B cells with the combined effects of antagomir-155 and hucMSCs-Exo. At the same time, ERK levels did not change significantly (Figure 5(b)). These results suggest that the inhibition of miR-155 expression level attenuates the ERK signaling pathway by reducing the activation of ERK. The recovery of SHIP-1 activity can promote B cell apoptosis, inhibit B cell proliferation, prevent B cell overactivation, and reduce inflammation in SLE patients by inhibiting ERK/SHIP-1 signaling pathway, thereby slowing down the disease. These data suggest that the miR-155 SHIP-1 axis may also be essential in regulating B-cell activation and survival.

### 3.6. Inhibiting B Cell Activation and Inflammation by Upregulating the Target Genes of miR-155

**Figure 6:**
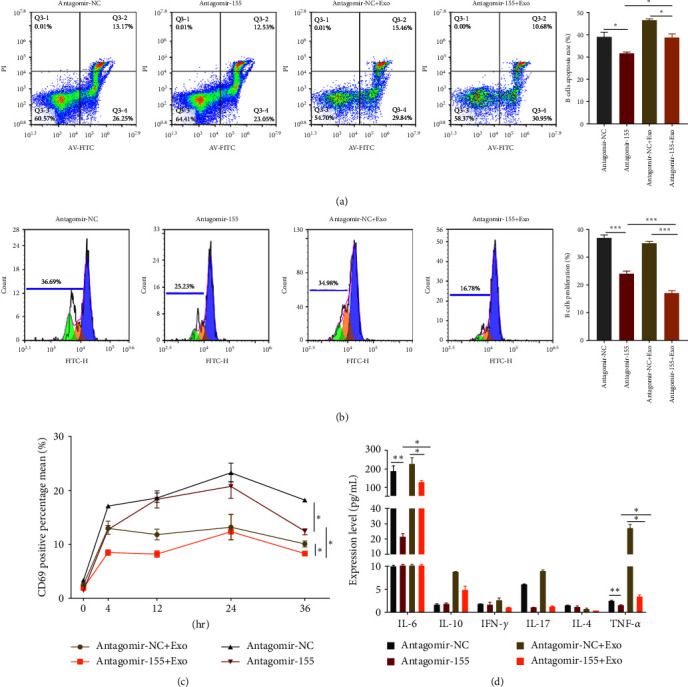
Inhibiting B cell activation and inflammation by upregulating the target genes of miR-155. (a) B cell apoptosis was detected by Annexin V-FITC after transfection of antagomir-155 and antagomir-NC. (b) SCFE was used to detect the proliferation of B cells after transfection of antagomir-155 and antagomir-NC. (c) antagomir-155 and hucMSCs-Exo were cocultured with PBMC cells, and B cells were sorted by CD19. Then the activation of 0–36 hr B cells was detected by flow cytometry using CD69. (d) antagomir-155 and hucMSCs-Exo were cocultured with PBMC for a certain time. After centrifugation, the supernatant was collected, and the cytokine levels were detected by multipellet flow immunofluorescence luminescence assay. Exosomes were isolated from hucMSC. Data are expressed as the mean ± SEM.  ^*∗*^*P* < 0.05,  ^*∗∗*^*P* < 0.01,  ^*∗∗∗*^*P* < 0.001.

To determine whether miR-155 promotes B cell apoptosis, inhibits proliferation, prevents excessive activation of B cells, and inhibits inflammation level, we transfected B cells with antagomir-155 (a miR-155 inhibitor) and compared with antagomir negative control (NC) (Figure 6(a)), inhibition of miR-155 promoted B cells apoptosis as measured by flow cytometry with Annexin V-FITC Apoptosis kit. To further verify the effect of hucMSCs-Exo and miR-155 inhibitor, B cells were incubated with hucMSCs-Exo at high concentration in the presence of antagomir-155, which promoted the apoptosis of B cells compared with the negative control. Antagomir-155 synergies with hucMSCs-Exo and promotes B cell apoptosis better. Similarly, the proliferation of B cells was detected by flow cytometry with an SCFE kit. The inhibition of miR-155 could promote the proliferation of B cells, and antagomir-155 synergized with hucMSCs-Exo had a better effect in promoting the proliferation of B cells (Figure 6(b)).

Next, we used CD69 to detect the overactivation of B cells. Flow cytometry results showed (Figure 6(c)) that the inhibition of miR-155 could inhibit the overactivation of B cells. The synergistic effect of the inhibitor and hucMSCs-Exo was more evident than the inhibitor alone. To determine whether miR-155 can inhibit the inflammation level of SLE patients, we transfected PBMC cells with antagomir-155, incubated them for a specific time, collected the cells, and centrifuged them. The supernatant was collected, and the cytokine was detected by multipellet flow immunofluorescence luminescence assay (IL-6, IL-10, INF-*γ*, IL-4, TNF-*α*) (Figure 6(d)). Compared with the control group, inhibition of miR-155 could reduce IL-6 and TNF-*α*. The synergistic effect of inhibitor and hucMSCs-Exo can also reduce IL-6 and TNF-*α* inflammation levels. Still, the synergistic effect is not as good as the effect of inhibiting miR-155 alone. This step needs to be further studied.

**Figure 7:**
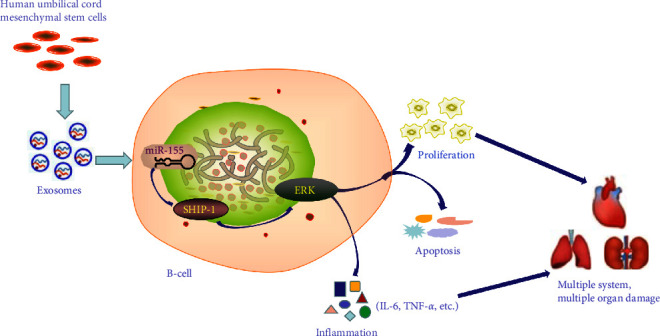
Schematic representation of immunomodulatory effects of hucMSCs-Exo on B cells from SLE patients. By coculture hucMSCs-Exo with B cells of SLE patients, B cells apoptosis can be promoted, B-cells proliferation can be inhibited, overactivation of B-cells can be prevented, inflammation can be alleviated, and disease progression can be slowed by reducing miR-155 targeting SHIP-1 in B cells.

Taken together, by coculture hucMSCs-Exo with B cells of SLE patients, B-cells apoptosis can be promoted, B-cells proliferation can be inhibited, overactivation of B-cells can be prevented, inflammation can be alleviated, and disease progression can be slowed by reducing miR-155-targeting SHIP-1 in B-cells (Figure 7).

## 4. Discussion

Variable groups have reported different results of lymphocyte subsets. For example, increased, decreased, or normal CD4+ and CD8+ T cell counts have been observed. However, the percentage of B cells is increased in almost all studies. Our data showed a decrease in CD4+ T cells and normal CD8+ T cells while an increase in CD19+ B cells in SLE patients. Hence, lymphocyte subsets are unbalanced in SLE patients, and abnormal B cell function is a critical player in the pathogenesis of SLE because autoantibodies are essential for diagnosis. In addition, B cells exhibit important immunological functions pertinent to SLE, such as the expression of autoantigens and the secretion of proinflammatory cytokines. Therefore, studying B lymphocytes can potentially unravel important pathogenic mechanisms of SLE and thus help develop more specific therapies to improve treatment efficacy and tolerability.

Whether hucMSCs-Exo regulates B lymphocytes in SLE patients is an interesting topic. Our *in vitro* coculture results showed that hucMSCs-Exo with a 50 *μ*g/mL concentration promoted B cell apoptosis, inhibited proliferation, and prevented overactivation and inflammation. The used dose of exosomes in most studies is approximately 10–100 *μ*g/mL. Our data showed that there was no significant difference between the lower dose (20 *μ*g/mL) and untreated PBMC (*P* > 0.05). No significant difference was observed in the inhibitory effect of 50 and 100 *μ*g/mL doses on B cell apoptosis. Additionally, doses higher than 100 *μ*g/mL were impractical because of inadequate exosome preparation. Based on the results, a series of concentration experiments are recommended in such research.

Another interesting topic is how to explain the immunomodulatory effects of hucMSCs-Exo on B cells from SLE patients. Recently, a study concentrated on the effect of exosome-derived miR-155 on acute lung inflammation demonstrated that exosomes mediate different biological functions and can regulate physiological and pathological processes *in vivo*. It is confirmed that miR-155 is a highly conserved and important miRNA that can regulate the inflammatory response, and the use of miR-155 inhibitors can significantly reduce inflammatory damage [19]. The pathogenicity of miR-155 is regulated by controlling the immune cell response. It has been shown that miR-155 significantly increases in whole spleen cells and spleen B and T cells in MRL-lpr mice, a mouse model of lupus [20]. In addition, miR-155 was also detected in SLE patients' serum and urine supernatant. Interestingly, the serum miR-155 level was higher than the liquid phase of the urine supernatant [21]. The rise or fall of miR-155 levels may be attributed to the effects of immunosuppressive drugs. Elevated levels of miR-155 have been reported in B cells in patients with SLE [22], but it is unclear whether miR-155 controls the expression of autoimmune responses and related pathologies. We found elevated levels of miR-155 in B cells of untreated SLE patients compared with healthy controls. This is consistent with previous reports, suggesting that elevated levels of miR-155 promote the pathogenesis of immune cells. Therefore, we hypothesized that hucMSCs-Exo inhibited miR-155 in lupus patients' B cells.

We further explored whether the regulation of miR-155 by exosomes could block the continuous activation of autoreactive B cells, thereby alleviating lupus-like diseases. We found that inhibition of miR-155 in B cells of SLE patients can promote B cell apoptosis, prevent B cell overactivation, and inhibit B cell proliferation. antagomir-155 had a better synergistic effect with hucMSCs-Exo, which confirmed that exosomes could regulate SLE autoimmunity.

MiRNAs are thought to regulate many complex physiological processes by inhibiting the expression of target genes with related functions [23]. KEGG analysis showed that miR-155 target genes were significantly enriched in immune and proliferation-related pathways. Through the database, we found that SHIP-1 is a potential target of miR-155. SHIP-1 is a tumor suppressor widely recognized to inhibit the proliferation of many tumor cells [10]. In terms of mechanism, we found that miR-155 partially controlled the expression of SHIP-1 in B cells, and the coaggregation of BCR and Fc*γ*RIIB led to the increase of SHIP-1 protein and impaired ERK activation of miR-155 in B cells. We found that under the combined effects of antagomir-155 and hucMSCs-Exo, p-ERK activation in B cells was inhibited compared with the control, while the ERK level was not significantly changed. These results suggest that the inhibition of miR-155 expression level attenuates the ERK signaling pathway by reducing the activation of ERK. The recovery of SHIP-1 activity can promote B cell apoptosis, inhibit B cell proliferation, prevent B cell overactivation, and reduce inflammation in SLE patients by inhibiting ERK/SHIP-1 signaling pathway, thereby slowing down the disease. These data suggest that the miR-155-SHIP-1 axis may also be essential in regulating B-cell activation and survival.

There are also some limitations in this paper. First, as SLE is an autoimmune disease easily affected by drugs or other factors, immune cell imbalance makes it more difficult to collect samples and separate and purify B cells, so labeled B cells after PBMC isolation are used for research. Second, exosomes have become potential biomarkers and therapeutic agents for autoimmune diseases in recent years, and further studies are needed to clarify what specific substances in exosomes have immunomodulatory properties. Therefore, continued research into the underlying mechanisms and applications will make this field more promising. Despite the rapid growth of exosome research, isolation, and purification techniques are still slow and irregular. Various techniques have been introduced, but these affect recovered exosomes' yield, diversity, and function. Once these limitations are overcome, new biomarkers can be identified to characterize exosomes and used for diagnostic applications. In addition, developing artificial exosome mimics with low side effects may be a new concept for achieving clinical-scale production of nanocarriers.

## 5. Conclusion

In conclusion, the application of exosomes offers excellent potential for preventing and treating human autoimmune diseases, especially SLE. Despite the above limitations, our study revealed that SHIP-1 regulates B-cell activation through the ERK signaling pathway, targeting miR-155 in B-cells and blocking the sustained activation of switch autoreactive B-cells, thereby alleviating lupus-like disease. Our results provide insight into how hucMSCs-Exo regulates autoimmunity in patients with lupus and suggest targeting miR-155 for autoimmunity while protecting immunity [24].

## Figures and Tables

**Table 1:** Primers sequences for qRT-PCR.

Primers	Sequences (5′ to 3′)
miR-155-5p F	GGGTTAATGCTAATCGTGAT
miR-155-5p R	CAGTGCGTGTCGTGGAGT
miR-155-5R T	GTCGTATCCAGTGCGTGTCGTGGAGTC GGCAATTGCACTGGATACGACACCCCT
SHIP-1 F	CATCTACACGCCTCTCACCC
SHIP-1 R	TCACGCTCCGTCTTCACAAA
U6 F	CTCGCTTCGGCAGCACA
U6 R	AACGCTTCACGAATTTGCGT
GAPDH F	GGAGTCCACTGGCGTCTTCA
GAPDH R	GTCATGAGTCCTTCCACGATACC

F, forward; R, reverse; RT, reverse transcription.

**Table 2:** Characteristics of untreated SLE patients and HC.

Features	HC (*n* = 22)	SLE (*n* = 28)	*P*-value
Age (years)	30.09 ± 4.06	31.86 ± 6.64	0.253
Gender (F/M)	16/6	20/8	0.919
Clinical manifestations			
Fever	0 (0.0%)	2 (7.1%)	0.201
Rashes	0 (0.0%)	6 (21.4%)	**0.021**
Arthritis	0 (0.0%)	7 (25%)	**0.011**
Serositis	0 (0.0%)	4 (14.3%)	0.065
Nephritis	0 (0.0%)	15 (53.6%)	<0.001
CNS involvement	0 (0.0%)	2 (7.1%)	0.201
Cardiac involvement	0 (0.0%)	3 (10.7%)	0.113
Gastrointestinal involvement	0 (0.0%)	4 (14.3%)	0.065

Laboratory data			
ANA antibody positive	0 (0.0%)	16 (57.1%)	<0.001
Anti-SSA antibody positive	0 (0.0%)	11 (39.3%)	<0.001
Anti-SSB antibody positive	0 (0.0%)	5 (17.9%)	<0.001
Anti-SM antibody positive	0 (0.0%)	6 (21.4%)	**0.002**
Anti-dsDNA antibody positive	0 (0.0%)	5 (17.9%)	**0.024**
WBC (×10^9^/L)	8.14 ± 1.36	6.14 ± 1.89	<0.001
Hb (g/L)	136.5 ± 9.93	118.43 ± 19.56	<0.001
PLT (×10^9^/L)	239.82 ± 15.27	196.61 ± 47.20	<0.001
C3 (g/L)	1.34 ± 0.12	0.43 ± 0.07	<0.001
C4 (g/L)	0.32 ± 0.35	0.13 ± 0.05	<0.001
24-hr urinary protein (g)	0.13 ± 0.02	3.74 ± 2.05	<0.001

Data are reported as means ± SD or number and percent within parentheses. CNS, central nervous system; ANA, antinuclear antibodies; dsDNA, double-stranded DNA; WBC, white blood cells; Hb, blood hemoglobin; PLT, blood platelets; C3, complement 3; C4, complement 4. Significant differences (*P* < 0.05) are shown in bold. Student's *t*-test, or the *χ*^2^ test, was used as appropriate.

## Data Availability

The data used to support the findings of this study are included in the article. Any other required data are available from the corresponding author or first author on reasonable request. miR-155 targets were predicted by TargetScan (https://www.targetscan.org/cgi-bin/targetscan/mamm_31/view_gene.cgi?taxid=9606&gs=INPP5D&members=miR-155). The SHIP-1-related genes were screened for GO (https://metascape.org/gp/index.html#/main/step1) analysis and showed to be related to the MAPK/ERK pathway.
